# Hyperbaric oxygen therapy for traumatic brain injury

**DOI:** 10.1186/2045-9912-1-21

**Published:** 2011-09-06

**Authors:** Lei Huang, Andre Obenaus

**Affiliations:** 1 Department of Biophysics & Bioengineering, Loma Linda University, Griggs Hall, Room 227, 11065 Campus St., Loma Linda, California, 92354, USA; 2 Department of Pediatrics, Loma Linda University, CSP A1010, 11175 Campus St., Loma Linda, California, 92354, USA; 3 Department of Radiology, Loma Linda University Medical Center, CSP A1010, 11175 Campus St., Loma Linda, California, 92354, USA; 4 Department of Radiation Medicine, Loma Linda University, CSP A1010, 11175 Campus St., Loma Linda, California, 92354, USA; 5 Department of Neuroscience, University of California, Riverside, 1140 Batchelor Hall, University of California, Riverside, California, 92521, USA

**Keywords:** intracranial pressure, metabolism, apoptosis, inflammation, tissue oxygenation, cerebral blood flow

## Abstract

Traumatic brain injury (TBI) is a major public health issue. The complexity of TBI has precluded the use of effective therapies. Hyperbaric oxygen therapy (HBOT) has been shown to be neuroprotective in multiple neurological disorders, but its efficacy in the management of TBI remains controversial. This review focuses on HBOT applications within the context of experimental and clinical TBI. We also discuss its potential neuroprotective mechanisms. Early or delayed multiple sessions of low atmospheric pressure HBOT can reduce intracranial pressure, improve mortality, as well as promote neurobehavioral recovery. The complimentary, synergistic actions of HBOT include improved tissue oxygenation and cellular metabolism, anti-apoptotic, and anti-inflammatory mechanisms. Thus HBOT may serve as a promising neuroprotective strategy that when combined with other therapeutic targets for TBI patients which could improve long-term outcomes.

## Introduction

Hyperbaric oxygen therapy (HBOT) is a treatment by which 100% oxygen is administered to a patient at a pressure greater than atmospheric pressure at sea level (i.e. one atmosphere absolute, ATA) [1]. The increased partial pressure of oxygen (pO_2_) within the blood and subsequent improved mitochondrial metabolism/tissue oxygenation constitutes the net effect of HBOT [2-6]. Given that the dissolved oxygen content in the plasma increases linearly after hemoglobin is 100% saturated [7,8], plasma bound oxygen can be used more readily than that bound to hemoglobin which enables tissue oxygen delivery even in the absence of red blood cells [7,9].

Thus, HBOT induces a much larger oxygen-carrying capacity in the blood that dramatically increases the driving force of oxygen diffusion to tissues. Although HBOT-induced cerebral vasoconstriction appears to be undesirable within the context of ischemic conditions [10,11] this may not be necessarily deleterious due to increased oxygen availability to injured tissues. HBOT may also counter vasodilation of the capillaries within hypoxic tissues, thereby minimizing collection of extravascular fluids (edema) which ultimately reduces brain vasogenic edema and the ensuing decrease in intracranial pressure (ICP) [5,12-14].

Emerging evidence has shown the neuroprotective effects of HBOT in a range of multiple injuries and/or disorders (Additional file 1, Table S1) [15]. The most common clinical applications include decompression sickness, carbon monoxide poisoning, minimization of radiation therapy induced tissue damage and enhancing skin grafts [1,16], which are all covered by insurance/Medicare. There are numerous "unapproved" uses of HBOT that focus on more complex neurological disorders, including autism, multiple sclerosis and stroke, which have shown promising results in experimental settings, but clinical efficacy is still elusive. Recent efforts have applied HBOT to traumatic brain injury [5,14,17]. While significant research on HBOT therapy has been undertaken (> 10,000 citations on PubMed), very little has been reported for HBOT within the setting of TBI (< 30 citations). We now briefly review the experimental and clinical HBO research relevant to TBI.

### HBOT in animal models of TBI

Early experimental research focused on the effects of HBO on brain edema, ICP and cerebral blood flow (CBF). Dunn and colleagues first demonstrated the neuroprotective effects of hyperoxia in a dog freeze-lesion model of brain injury that simulated a brain contusion. Hyperoxia significantly improved outcomes by reducing mortality [18]. Reduced ICP (30% decrease) and CBF (19%) were also reported in a dog model of brain injury treated by HBOT (2 ATA for 4 hrs) [19]. The absence of changes in cerebrospinal fluid (CSF) lactate, a marker of brain injury, following HBOT further supported the notion that HBO improved tissue oxygen delivery despite the undesirable decrease in CBF subsequent to vasoconstriction [10]. Expanding on the original model, various methods (psyllium seed, extradural balloon) were used to induce brain edema in dogs followed by HBOT [20-23]. Using HBOT at 3 ATA for 45 min [20,21] or at 2 ATA for 4 hrs [22] resulted in a significant decrease (> 50%) in mortality relative to non-treated injured animals. They also reported significantly less brain edema [20] and reduced cisternal CSF pressure [21] in the HBOT groups. Sukoff and colleagues suggested that the improvement seen in their model was due to the effectiveness of HBOT against ischemia, secondary to the induced cerebral edema [21]. Hayakawa et al, however, found that HBOT (3 ATA for 1 hr) did not or barely changed CSF pressure and CBF in most injured dogs [23].

In a rat model of moderate fluid percussion injury, the neuroprotection afforded by HBOT translated into long term cognitive improvements, characterized by a shorter latency to find a hidden platform in Morris water maze (MWM) performance [2]. Within brain tissues, HBOT showed significant protection against hippocampal neuronal loss compared to normobaric oxygen treatment [2]. Importantly, there was no increased free radial peroxide and peroxynitrite production, suggesting the absence of oxygen toxicity after HBOT [2]. Studies in a model of cerebral ischemia concurred that HBOT did not exacerbate lipid peroxidation [24].

The aforementioned neuroprotective efficacy of HBOT was all achieved when intervention was administered during the acute phase (within hours) of post-TBI. The prolonged therapeutic time window of HBOT was further investigated in studies using a focal cortical weigh-drop model of TBI [25-27]. Wang and colleagues have demonstrated that multiple HBOT (3 ATA hourly for 3 or 5 days), delivered at latest 2 days post-injury resulted in significantly reduced overall neurological deficit scores and neuronal apoptosis within brain tissue. But the authors also showed that twelve hours post-TBI is the latest effective window for neuroprotection when a single episode of HBOT was delivered [27]. Moreover, Harch et al [25,26] have tested the effects of low pressure HBOT (90 min twice a day at 1.5 ATA) which started at 50 days after the initial brain injury for a total duration of 40 days. At end of the treatment (100 days post injury), MWM spatial learning performance in the HBOT groups improved significantly and was highly correlated with increased ipsilateral hippocampal blood volume (cerebrovascular density) measured by diaminobenzadine blood stain [25,26]. Given the well-described presence of angiogenesis in HBOT in other brain injury models [28,29], the authors suggested that angiogenesis was the most likely explanation for the HBOT-induced recovery of function. They claimed that coupling of "blood flow and metabolism" and "metabolism and function" were potential mechanisms, as both were increased in animals receiving HBOT. This hypothesis is consistent with the pattern of HBOT-induced increases in blood flow seen on single photon emission computed tomography brain imaging in patients with chronic TBI [30,31]. A caveat is that HBOT failed to improve forelimb placing function, likely due to the reported tissue loss within the sensorimotor cortex following TBI [26].

**Figure 1:**
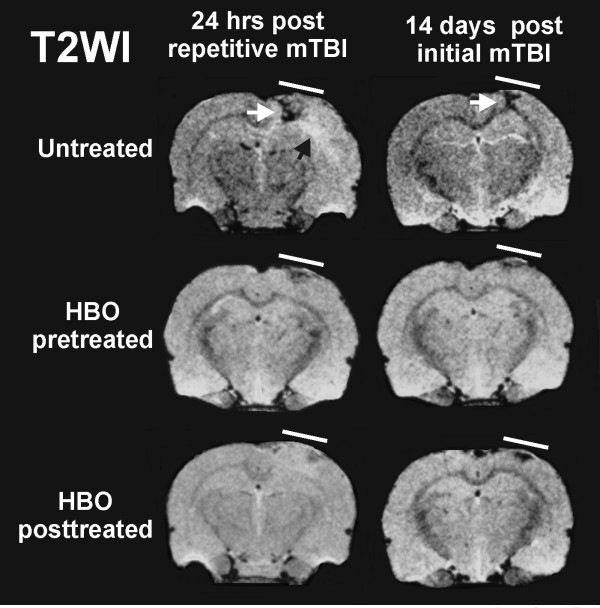
**HBO reduces rmTBI lesion volumes**. Pre- and post-treatment with HBO reduces lesion volume identified from magnetic resonance imaging (MRI, T2 weighted images). Repetitive mild traumatic brain injury (rmTBI) was induced 3 days apart and resulted in ipsilateral tissue damage. On T2WI, hypointensities (white arrow) are consistent with bleeding while hyperintensities (black arrow) suggest edema formation. At 24 hrs after the rmTBI, HBO pre- or post-treatment significantly reduced the lesion size compared to untreated animals. The neuroprotective effects persisted to 14 days after the initial mTBI.

**Figure 2:**
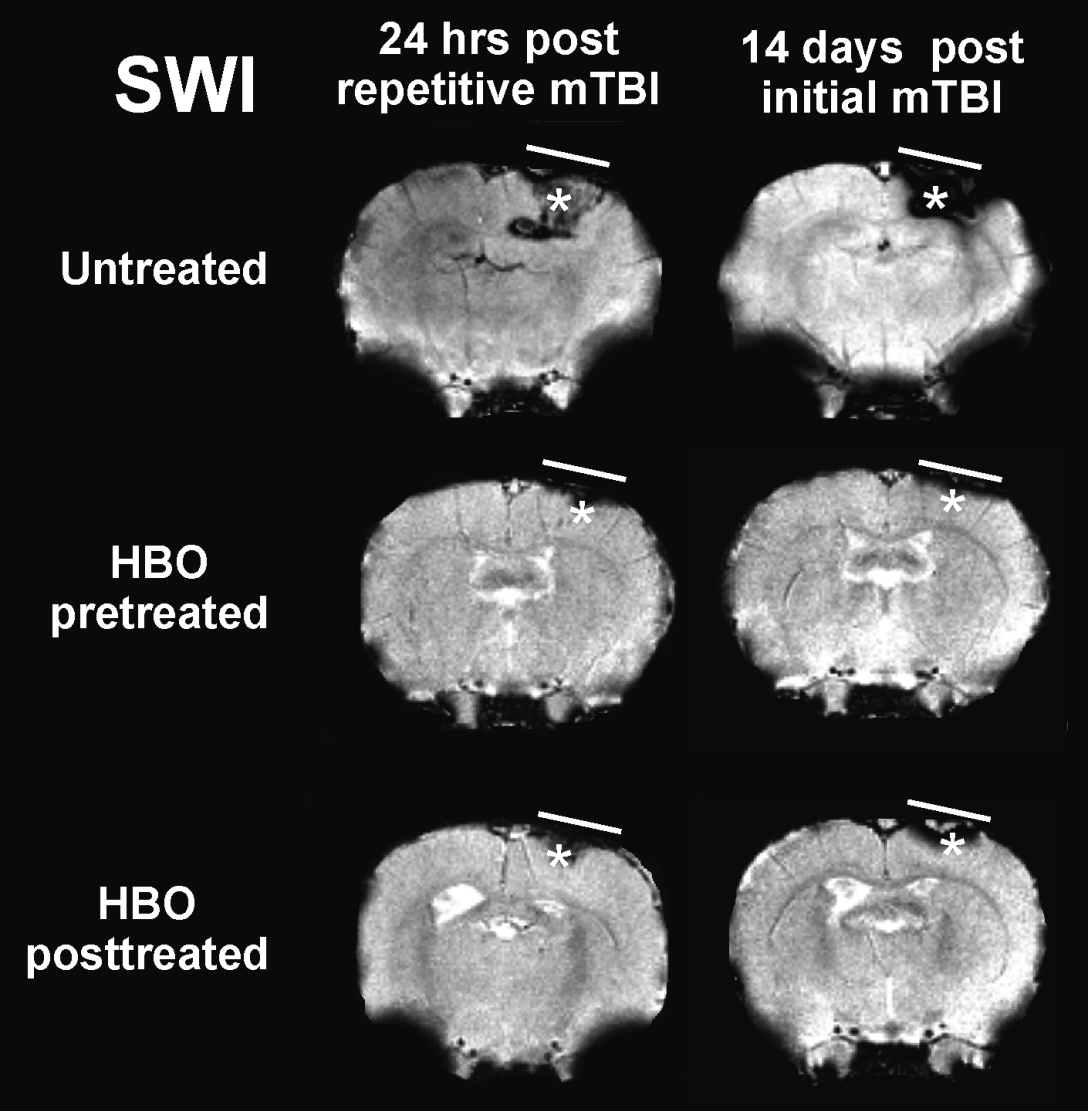
**HBOT reduces extravascular blood after rmTBI**. HBO pre- and post-treatment improved susceptibility weighted imaging (SWI)-identified intracerebral hemorrhage following repetitive mild traumatic brain injury (rmTBI) 3 days apart. At 24 hrs after rmTBI, HBO pre- or post-treatment significantly ameliorated the hemorrhage (hypointensity, asterisks) compared to untreated animals, which persisted to 14 days after the initial mTBI.

We recently investigated both prophylactic (pre-treatment) hyperbaric oxygen (HBO) strategy and HBOT (post-treatment) for treatment of repetitive mild traumatic brain injury (rmTBI) (personal communication: Drs. Lei Huang and Andre Obenaus). Repetitive mTBI is an important public health concern for sports athletes and active military personnel as subsequent injuries are thought to exacerbate existing neuropathology. Mild controlled cortical impact (CCI) was used to model rmTBI in adult rats. In rmTBI animals, a second mild CCI was delivered at the same location at 3 or 7 days after the initial impact. HBO pre-treatment or HBOT was given 1 hr daily at 2 ATA for 3 consecutive days either prior to or 24 hrs after the initial TBI, respectively. T2 weighted imaging (T2WI) and susceptibility weighted imaging (SWI) were acquired non-invasively from which lesion and hemorrhage volumes were quantified. Our results clearly demonstrated that both HBO pre-treatment and HBOT improved neuroimaging outcomes following rmTBI, in contrast to those seen in tissues without HBO intervention. There were significant reductions in the T2WI-derived lesion and SWI-identified hemorrhage volumes at 24 hrs after rmTBI (Figures 1, 2). The most dramatic neuroprotective effects were observed in animals receiving rmTBI 3 days apart where a 3-fold reduction in hemorrhage volumes was observed compared to Shams (Figure 2). Given that the pathophysiological processes underlying rmTBI likely involves cellular metabolic perturbations in the injured brain [32], a neuroprotective approach, namely, HBO pre-treatment or HBOT favoring cerebral aerobic metabolism could be beneficial. Similar findings have been reported in HBOT for human severe TBI [14].

### Clinical HBOT for human TBI

A variety of human injuries and neurological diseases have applied HBOT to improve outcomes. More overt neurological injuries, such as stroke or TBI have not been aggressively pursued, partly due to apparent or perceived contraindications. While a complete listing of the absolute and relative contraindications for clinical applications are beyond the scope of this review, it should be noted that certain drugs, fever and respiratory ailments limit clinical application of HBOT. However, within the realm of clinically applied HBOT for TBI very little research has been conducted. Enthusiasm for HBOT for brain injured patients was dampened by the findings of a meta-analysis of TBI patients receiving HBOT [33]. Their sobering conclusions were that the risk of death was reduced but there was no apparent change in clinical outcomes. However, as these authors acknowledged, variance in treatment protocols and the limited number of patients in the studies reviewed hampered their analysis. Based on their findings they suggested that HBOT could not be justified for TBI patients.

The poor clinical outcomes of earlier HBOT studies combined with the relative success of normobaric oxygen therapy (NBOT) in TBI have lead some to propose that normobaric oxygen therapy should be used preferentially in brain-injured patients. There are numerous studies that demonstrate an enhanced clinical outcomes by treatment with normobaric oxygen [34]. Much of the enthusiasm for use of NBOT is based on a prospective study of severe TBI patients [4]. Narotam and colleagues [35] evaluated brain tissue oxygen concentrations in patients with severe TBI. Using Licox oxygen probes, 139 patients were studied using a pO_2 _protocol that maintained brain oxygen levels to > 20 mm Hg. They elegantly demonstrated that normobaric oxygen therapy significantly reduced mortality, but moreover, they showed improved clinical outcomes at 6 mo post-severe TBI. A similar study found that hyperoxia improved the cerebral metabolic rate of oxygen in severe TBI patients using O15-postiron emission tomography, but they did not compare to HBOT treated patients [36]. Thus, at a minimum, NBOT could be beneficial for TBI patients.

The most extensive research into clinical application of HBOT for TBI patients has been conducted by the Rockswolds [5,14,17,37]. Almost 30 years ago they undertook one of the first clinical trials in evaluating the benefits of HBOT for severe head injured patients [17]. In that early study they demonstrated a 50% decrease in mortality but found no changes in the clinical outcome status (i.e. good recovery and moderate disability). As noted above, a meta-analysis of several studies concluded the same findings; reduced mortality but no change in clinical status [33].

While survival was increased, functional recovery was not changed, leading to questions about the timing of the HBOT. In addition, the mechanism(s) underlying HBOT and its effects on cerebral metabolism had not been previously established in severely brain-injured patients. In another prospective clinical trial, Rockswold et al [5], reported increased cerebral metabolic rate of oxygen and decreased lactate measured from cerebrospinal fluid after HBOT (1.5 ATA 1 hr/day every 24 hrs with a maximum of seven sessions). ICP was also reduced but a caveat was noted that these changes did not last till the next session [5]. It also serves to remind the reader that similar results had also been previously reported in NBOT [36].

Based on evidence that NBOT of human TBI patients appeared to have similar outcomes as patients who underwent HBOT, a follow-up study was conducted to compare these two groups after severe TBI to assess the efficacy of therapy [14]. It is important to note, that the standard of care is neither NBOT nor HBOT. Thus, their study design included controls (standard of care), normobaric (3 hrs 100% O_2_) and HBOT (1.5 ATA for 60 min) that received their initial treatment within 24 hrs of a severe TBI. Treatments were then conducted daily for 3 consecutive days. The pO_2 _levels within the brain were nearly 3 fold higher in the HBOT compared to the NBOT groups and significantly different from controls [14]. As previously reported, they found increased cerebral metabolic rate of oxygen, decreased lactate and decreased intracranial pressure. They also reported that HBOT increased cerebral blood flow. Perhaps the most important finding was that an indicator of mitochondrial dysfunction, lactate/pyruvate ratios, were significantly decreased only in the HBOT group. They also demonstrated no adverse outcomes or harmful effects in patients receiving HBOT [14]. Thus, HBOT for severe TBI, appears to improve cellular survival which was not observed in NBOT group.

Based on these limited studies, it is clear, that HBOT could be an effective therapy for clinical severe TBI. Compared to NBOT, HBOT assists in improving brain "functions", such as cerebral metabolism and blood flow. However, additional studies are needed not only during the acute phase of the injury, but also long-term studies evaluating outcomes to determine if HBOT is beneficial to TBI patients.

### Putative mechanisms underlying the neuroprotection of HBOT following TBI

Over the past several decades, the neuroprotective mechanisms of HBOT have been investigated in a variety of animal models of TBI. The initial work in dogs (see above) have shown the HBOT was able to increase tissue oxygen delivery [10] as well as to protect penumbra tissue from secondary ischemia [21]. Based on the dog model, a similar freeze-induced brain injury was conducted in rats to evaluate local cerebral glucose utilization using the autoradiographic 2-deoxyglucose technique. Compared to animals that underwent NBOT, a four-day HBOT course (2 ATA for 90 minutes daily) significantly reversed the depressed glucose utilization within gray matter ipsilateral to the lesion [38]. Interestingly, HBOT tended to decrease glucose utilization in the sham-operated animals. However, it was still uncertain whether the favorable outcomes were directly attributable to improved glucose metabolism associated with HBOT. HBO-improvements in tissue oxygenation and mitochondrial metabolic function were further investigated in a rat model of fluid percussion injury (FPI) [3]. HBOT (1 hr 1.5 ATA with 3 hrs 100% normobaric oxygen) treatments significantly improved, 1) brain tissue pO_2 _(more than 6 fold) near the site of injury; 2) ex vivo brain tissue oxygen consumption (vO_2_, more than 1 fold); and 3) recovery of synaptosomal mitochondrial metabolic activity [39,40].

Given that the prognosis of TBI clearly depends on the processes of cell death and survival that occur within the traumatized tissues, neuroprotective therapies need to mitigate and improve survival and function within the remaining viable perilesional brain tissue [41]. The neuroprotective effects of HBOT against secondary brain damage in the penumbra region have been extensively investigated [6,41-43]. Using a model of dynamic cortical deformation (DCD) to produce focal cerebral contusion in rats, HBOT (2 sessions at 2.8 ATA for 45 min/each) were administered at 3 hrs after TBI and compared to the effects of NBOT. There were significantly smaller lesion volumes and decreased numbers of terminal deoxynucleotidyl transferase dUTP nick end labeling (TUNEL, a biomarker for apoptosis) positive cells after HBOT. Normobaric oxygen therapy (100%) also improved tissue measures but not to the extent found after HBOT [43]. The anti-apoptotic modulator, B-cell lymphoma (Bcl-2), was increased after HBOT and correlated to reduced tissue apoptosis [41]. Similar changes were found for B-cell lymphoma-extra large (Bcl-xl) expression, while the pro-inflammatory protein, B-cell lymphoma-associated X protein (Bax), was observed primarily in astrocytes instead of neurons. The ratio between pro-apoptotic Bax and anti-apoptotic Bcl-2/Bcl-xl proteins has been shown to act as a "rheostat" that sets the threshold [44] of susceptibility to apoptosis by competitively modulating the opening of the mitochondrial permeability transition pore (mPTP) [45,46]. Enhanced Bcl-2 expression inhibits the mPTP that subsequently preserves mitochondrial homeostasis and therefore the integrity of the electron transport chain [6]. Palzur et al thus hypothesized that HBOT-induced increases in Bcl-2 expression and the resultant increase in intracellular oxygen bio-availability may contribute both to preserve mitochondrial integrity and to reduce the activation of the mitochondrial mediated apoptotic pathway following TBI [6]. In the same animal model, HBOT (2.8 ATA for 45 min at 3 and 24 hrs post-injury) substantially facilitated the recovery of mPTP expression. Subsequently, TBI-induced injury to tissue morphology was reversed with enhanced neuronal survival and preserved axonal architecture within perislesional tissues [6]. Similar findings of improved mitochondrial redox after HBOT in the FPI model of TBI have also been reported [3]. The preservation of mitochondrial integrity by HBOT hindered the activation of mitochondria-associated apoptotic pathways by significantly lowering caspase 3 and 9, but not caspase 8 expression (critical for non-mitochondrial apoptotic pathway) in injured brain tissues [6]. These results underscore the importance of HBOT-induced reductions in delayed cell death within the tissue penumbra after TBI. Such, mechanisms echo the neuroprotection of HBO seen in brain ischemia and subarachnoid hemorrhage [47,48].

Acute inflammation also plays an important role in secondary brain damage after TBI. An influx of inflammatory cells induced by TBI provides the primary source of matrix metalloproteinases (MMPs) activity [49]. MMPs in the injured brain further play a deleterious role and promote cell death, including apoptosis [50]. The effects of HBOT on inflammatory infiltration and the expression of (MMPs) have been explored in a rat model of DCD. Both HBOT (2.8 ATA for 45 min at 3 hrs after injury and twice a day thereafter for 3 consecutive days) and NBOT significantly decreased myeloperoxidase-positive neutrophils within the traumatic penumbra, but HBOT had a more pronounced effect. HBOT also significantly reduced the elevation of MMP-9 expression associated with neutrophilic infiltration [42]. Thus, HBOT substantially decreases the harmful effects of inflammation by reducing MMP-9 over-expression that then results in a reduction of delayed cell death in penumbral tissues surrounding the site of injury. Interestingly, reduced MMP-9 has also been proposed to be the underlying mechanism associated with HBO pretreatment induced neuroprotection against TBI at high altitude in a rat model [51]. However, what is still unresolved is whether the decreased numbers of apoptotic cells following HBOT, is a direct anti-apoptotic effect or secondary consequence due to HBOT anti-inflammatory effects. Further studies are warranted to disclose the complex mechanisms underlying the neuroprotective effects of HBOT after TBI.

## Conclusions

Translational research of HBOT in a variety of TBI models has shown neuroprotective effects in the absence of increased oxygen toxicity when administered at pressures less than 3 ATA. Due to the heterogeneity of human TBI, the efficacy of clinical HBOT and an optimal regimen for HBOT remains elusive. However, all human studies have involved severe TBI patients and it is likely that there may be increased efficacy in mild or moderate TBI patients. Recent clinical trials favor HBOT as promising safe therapeutic strategy for severe TBI patients. Although both NBOT and 1.5 ATA HBOT can be neuroprotective, HBOT exerts more robust and long-lasting effects in the absence of pulmonary or cerebral oxygen toxicity. The improved tissue oxygenation and cellular metabolism, anti-apoptotic as well as anti-inflammatory effects may constitute the multiple and complementary mechanisms underlying HBOT-induced neuroprotection.

## List of abbreviations

ATA: One atmosphere absolute; Bax: B-cell lymphoma-associated X protein; Bcl-2: B-cell lymphoma; Bcl-xl: B-cell lymphoma-extra large; CBF: Cerebral blood flow; CCI: Controlled cortical impact; CSF: Cerebrospinal fluid; DCD: Dynamic cortical deformation; FPI: Fluid percussion injury; HBO: Hyperbaric oxygen; HBOT: Hyperbaric oxygen therapy; ICP: Intracranial pressure; MMPs: Matrix metalloproteinases; mPTP: Mitochondrial permeability transition pore; MWM: Morris water maze; NBOT: Normobaric therapy; pO_2_: Partial pressure of oxygen; rmTBI: Repetitive mild traumatic brain injury; SWI: susceptibility weighted imaging; TBI: Traumatic brain injury; TIMP-1: metallopeptidase inhibitor-1; TUNEL: Terminal deoxynucleotidyl transferase dUTP nick end labeling; T2WI: T2 weighted imaging; vO_2_: Oxygen consumption

## Competing interests

The authors declare that they have no competing interests.

## Authors' contributions

Both LH and AO contributed intellectually to this review. LH reviewed the HBO studies in animal models of TBI and AO reviewed clinical trials of HBO in severe TBI patients. All authors read and approved the final manuscript.

## Supplementary Material

Additional file 1 **Table S1: Current clinical uses for HBOT**. Click here for file
