# Supplementary material for: Hyperbaric oxygen therapy for traumatic brain injury
Source: Med Gas Res. 2011 Sep 6;1:21. doi: 10.1186/2045-9912-1-21 (PMC3231802; doi:10.1186/2045-9912-1-21)
Supplement: Additional file 1 — Table S1: Current clinical uses for HBOT. [file 2045-9912-1-21-S1.DOC]

**Table S1:** Current clinical uses for HBOT

| **Insurance or Medicare Reimbursed** | **Off-label/Non-Insurance** |
| --- | --- |
|  |  |
| Air or Gas Embolism | Autism |
| Carbon Monoxide Poisoning | Cerebral Palsy |
| Compartment Syndrome/Crush Injury/Other Traumatic Ischemia | Lyme Disease |
| Decompression Sickness (Bends) | Migraine |
| Diabetic and Selected Wounds | Multiple Sclerosis |
| Exceptional Blood Loss (Anemia) | Near Drowning |
| Gas Gangrene | Recovery from Plastic Surgery |
| Intracranial Abscess | Sports Injuries |
| Necrotizing Soft Tissue Infection | Stroke |
| Osteoradionecrosis and Radiation Tissue Damage | **Traumatic Brain Injury** |
| Osteomyelitis (Refractory) |  |
| Skin Grafts and (Compromised) Flaps |  |
| Thermal Burns |  |

*Modified from Edwards ML, 2009*
